# ABO blood group associated with cerebral venous thrombosis after Oxford-AstraZeneca COVID-19 vaccination: a case–control study

**DOI:** 10.1177/01410768231214341

**Published:** 2023-12-12

**Authors:** Gie Ken-Dror, Pankaj Sharma, on behalf of the international Bio-Repository to Establish the Aetiology of Sinovenous Thrombosis (BEAST) collaborators and Cerebral Venous Sinus Thrombosis With Thrombocytopenia Syndrome Study Group, Anita van de Munckhof

**Affiliations:** 1 Institute of Cardiovascular Research Royal Holloway, University of London (ICR2UL), London TW20 0EX, UK; 2 Department of Clinical Neurology, Imperial College Healthcare NHS Trust, London W6 8RF, UK

**Keywords:** ChAdOx1-S COVID-19 vaccine, Oxford-AstraZeneca, cerebral venous thrombosis, blood group

## Abstract

**Objectives:**

To determine whether blood group influences development of cerebral venous thrombosis (CVT) after administration of the coronavirus disease 2019 (COVID-19) AstraZeneca ChAdOx1-S vaccine.

**Design:**

A case–control study. Univariate and multivariate logistic regression was used to determine the association between blood type and COVID-19 vaccination status.

**Setting:**

Vaccinated and unvaccinated patients recruited from the international Bio-Repository to Establish the Aetiology of Sinovenous Thrombosis study and the Cerebral Venous Sinus Thrombosis With Thrombocytopenia Syndrome Study Group.

**Participants:**

All patients were of European descent and age and sex matched. Cases (*n* = 82) were patients ≥18 years old who suffered a CVT within 28 days of a first dose of ChAdOx1-S vaccine. Controls (*n* = 441) were unvaccinated CVT patients ≥18 years old. All patients were of European descent.

**Main outcome measures:**

Frequency of blood type and ABO allele distribution by vaccination status.

**Results:**

Blood group O was found to be more prevalent among CVT patients with vaccine-induced thrombotic thrombocytopenia (VITT-CVT) after ChAdOx1-S vaccination compared with unvaccinated CVT cases (43% vs. 17%, respectively, *p* < 0.001). Blood group A was less prevalent, though still high, in the vaccinated group compared with the unvaccinated group (47% vs. 71%, respectively, *p* < 0.001). No significant differences were observed in the VITT-CVT non-ChAdOx1-S vaccine group and unvaccinated pre-COVID-19 CVT group for blood group.

**Conclusions:**

Blood group O is more prevalent among patients with VITT-CVT after ChAdOx1-S vaccination compared with unvaccinated cases, independent of well-established CVT risk factors. A larger dataset may be able to determine whether those of blood groups B and/or AB may be safely vaccinated with the low cost, readily available and easily transported ChAdOx1-S rather than adopting a complete ban.

## Introduction

Cerebral venous thrombosis (CVT) has been associated with the ChAdOx1-S (Oxford-AstraZeneca) coronavirus disease 2019 (COVID-19) vaccine,^1–3^ leading many countries to limit its use in younger individuals, despite its high efficacy, cost-effectiveness and ease of handling. Identifying individuals most likely at risk of this rare adverse event would allow a more targeted restriction of this inexpensive vaccine. After administration of ChAdOx1-S, CVT has been reported within 28 days of vaccination and associated with a distinct clinical profile (which includes thrombosis with thrombocytopenia syndrome) and high mortality rate.^4^ Most of these CVT cases after vector-based COVID-19 vaccination are due to vaccine-induced thrombotic thrombocytopenia (VITT),^5^ often with a poorer outcome.^6^

Recent studies have examined the relationship of blood group with COVID-19.^7^ A genome-wide association study (GWAS) of COVID-19 patients showed that individuals with blood group A had a higher risk of severe disease (odds ratio [OR] = 1.45), while individuals with B or O blood groups were less represented among COVID-19 patients.^8^ In addition, critically ill COVID-19 patients with blood groups A and AB were more likely to require mechanical ventilation and prolonged intensive care compared with patients with blood groups B or O.^8^ We reported the first GWAS in patients with CVT^9^ finding that non-O blood group is associated with a 3-fold (*p* = 10^−16^) increase of CVT compared with individuals with blood type O.

With increasing evidence that onset and outcome after CVT is associated with blood group, we sought to determine whether blood group influences development of CVT after AstraZeneca ChAdOx1-S vaccination.

## Methods

All 82 patients who suffered their first VITT-CVT (47 of which were defined using the international classification of *Definite* or *Probable*)^5^ within 28 days of a first dose of ChAdOx1-S vaccine from the Cerebral Venous Sinus Thrombosis with Thrombocytopenia Syndrome Study Group,^4^ and 441 pre-COVID-19 unvaccinated patients with CVT from the Bio-Repository to Establish the Aetiology of Sinovenous Thrombosis (BEAST) study were recruited. The BEAST consortium, located in nine research centres (Belgium, Finland, Italy, the Netherlands, Portugal, Sweden, France, UK and USA), was established to analyse the epidemiology and genetics of CVT.^9,10^ Diagnoses were imaging confirmed using computed tomography/venography, magnetic resonance or conventional angiogram. Both cohorts are described in detail elsewhere^4,9,10^ but briefly, all patients were of European descent and age and sex matched (Table 1).

**Table 1.** Characteristics of vaccinated and unvaccinated patients with CVT.

	Vaccinated (*n* = 82)	Unvaccinated (*n* = 441)	*p*-Value
Age in years at CVT (SD)	47.8 (15.1)	47.4 (15.4)	0.83
Women (*n*/%)	57 (69.5)	305 (69.2)	0.99
COVID-19 vaccination (*n*/%)			
First (1st)			
ChAdOx1-S vaccine	59 (72.0)	0 (0)	–
Non-ChAdOx1-S vaccine^a^	23 (28.0)	0 (0)	–
Second (2nd)			
None	67 (81.7)	0 (0)	–
ChAdOx1-S vaccine	2 (2.4)	0 (0)	–
Non-ChAdOx1-S vaccine^b^	13 (15.9)	0 (0)	–
Third (3rd)			
None	81 (99)	0 (0)	–
ChAdOx1-S vaccine	0 (0)	0 (0)	–
Non-ChAdOx1-S vaccine^c^	1 (1)	0 (0)	–
VITT (*n*/%) for ChAdOx1-S vaccine			
Definite	38 (64.4)	0 (0)	–
Probable	9 (15.3)	0 (0)	–
Possible	5 (8.5)	0 (0)	–
Unlikely	7 (11.9)	0 (0)	–
VITT (*n*/%) for *Non*-ChAdOx1-S vaccine			
Definite	4 (17.4)	0 (0)	–
Probable	0 (0)	0 (0)	–
Possible	3 (13.0)	0 (0)	–
Unlikely	16 (69.6)	0 (0)	–

*n*: sample size; SD: standard deviation; VITT: vaccine-induced immune thrombocytopenia and thrombosis.

^a^ Pfizer/BioNTech (12), Moderna (2), Johnson & Johnson (5), Sinovac (1), Other (3). ^b^ Pfizer/BioNTech (8), Moderna (2), Johnson & Johnson (0), Sinovac (0), Other (3). ^c^ Pfizer/BioNTech (1), Moderna (0), Johnson & Johnson (0), Sinovac (0), Other (0)

Vaccinated patients were free of these CVT risk factors: anticoagulant, antiphospholipid antibodies, Protein-C deficiency, Protein-S deficiency, antithrombin deficiency, Factor-V Leiden and factor VIII (FVIII), except one patient with prothrombin G20210A and one patient with lupus anticoagulant. Unvaccinated patients with CVT from the BEAST study were also free of those risk factors.

All recruits were aged ≥18 years with patient or relative informed written consent. Age was documented at time of CVT diagnosis.

Both studies received all ethical and consent standards set by local Institutional Review Boards at each of the participating sites.

## Statistical methods

Matching of vaccinated and unvaccinated patients was performed using the frequency matching approach. First, we calculated the distribution (frequency table) of the matching characteristics among the vaccinated patients. Characteristics were categorised as follows: age: ≤33 years, >33 and ≤43 years, >43 and ≤51 years, >51 and ≤62 years, >62 years and gender (men, women). For each category of the matching characteristic, we selected an unvaccinated patient from the BEAST study. If the unvaccinated patients matched on the age and sex category, the matching was considered successful and removed from the unvaccinated patient list. An iterative approach was applied if matching failed. Sample size was thus maximised using this iterative strategy until all categories were matched.

Descriptive statistics summarised data using mean with standard deviation or median with interquartile range for continuous variables, and proportion for categorical variables. For single factor analysis, chi-square (or Fisher’s exact test, where appropriate) was used for categorical variables, and independent *t*-test (or Mann-Whitney U test, where appropriate) for continuous variables. Univariate and multivariate logistic regression estimated the associations of covariates (confounding variables) with COVID-19 vaccination status. The reliability (goodness of fit) of each model was quantified using the Hosmer and Lemeshow test.^11^ Models were evaluated using Akaike's Information Criterion and the likelihood ratio chi-square test.^12^ ABO alleles distribution for vaccinated patients and unvaccinated patients was estimated by the Expectation-Maximization-algorithm from blood type distribution.^13,14^ A permutation test assessed significant differences in ABO allele frequencies between vaccinated and unvaccinated patients. Rather than using a correction for multiple testing at global significance level, the statistical significance threshold for individual association testing was established at *P*<0.01.^15^

## Results

A total of 82 CVT vaccinated patients and 441 non-vaccinated patients of European descent were enrolled in the study to assess whether certain ABO blood type group was more likely to be associated with development of CVT after the COVID-19 ChAdOx1-S vaccination. Of those, 47 patients were defined using the international classification for CVT post vaccination of *Definite* or *Probable* within 28 days of a first dose of ChAdOx1-S. The demographic characteristics of CVT individuals with COVID-19 vaccine and non-COVID-19 vaccine are presented in Table 1. Patients were appropriately age and sex matched. Importantly, there were no differences between VITT-CVT non-ChAdOx1-S vaccine group and unvaccinated pre-COVID-19 CVT group for well-established risk factors for CVT including lupus anticoagulant, antiphospholipid antibodies, Protein-C deficiency, Protein-S deficiency, antithrombin deficiency, factor VIII (FVIII), Factor-V Leiden and prothrombin G20210A.

**Figure 1.**
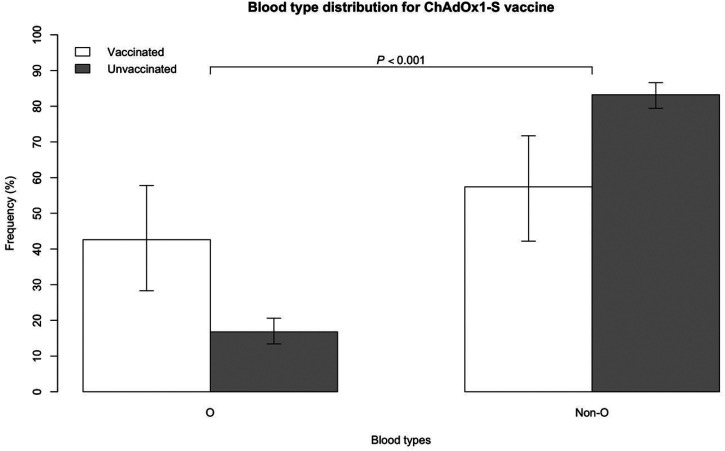
Blood type distribution between vaccinated group for ChAdOx1-S vaccine (analysis restricted to VITT-CVT defined as *Definite* or *Probable*^5^) and unvaccinated group.

Blood type distribution of VITT-CVT (*Definite* or *Probable*) vaccinated and unvaccinated groups are presented in Table 2. Blood group O was more prevalent in the VITT-CVT group than in the unvaccinated pre-COVID-19 CVT group (43% vs. 17%, respectively, *p* < 0.001, Figure 1). Blood group A was less prevalent, though still high, in the vaccinated compared with the unvaccinated group (47% vs. 71%, respectively, *p* < 0.001), as described in our previous GWAS.^9^ In an unadjusted model, the presence of blood group O was three-times more likely in the VITT*-*CVT *Definite* or *Probable* vaccinated group (OR = 3.83, 95% confidence interval (CI): 1.98–7.41, *p* < 0.001) compared with those with blood group A.

**Table 2.** Blood type distribution between the vaccinated group (analysis restricted to VITT-CVT defined as *Definite* or *Probable*^5^) and the unvaccinated group.

	Vaccinated(*n* = 47)	Unvaccinated (*n* = 441)	Overall*p*-Value
Blood type (*n*/%) for ChAdOx1-S vaccine^a^			
O	20 (42.6)	74 (16.8)	<0.001
A	22 (46.8)	312 (70.7)	
B	2 (4.3)	39 (8.8)	
AB	3 (6.4)	16 (3.6)	

*n*: sample size.

a Analysis includes only VITT defined as *Definite* or *Probable*.^5^

For the non-ChAdOx1-S vaccine group and unvaccinated pre-COVID-19 CVT, there was no significant difference between ABO blood groups between vaccine and unvaccinated patients. There was no significant difference in ABO blood groups between the 7 (Definite, Probable, Possible) non-ChAdOx1-S vaccinated and the 441 unvaccinated patients (*p* = 0.34). Similarly, when analysis is confined to only the Definite group (*n* = 4) there was no difference between vaccinated and unvaccinated patients, blood group O compared to Non-O (*p* = 0.52).

Multivariate analysis with well-established CVT risk factors lupus anticoagulant, antiphospholipid antibodies, Protein-C deficiency, Protein-S deficiency, antithrombin deficiency, factor VIII (FVIII), Factor-V Leiden (FAK-tur five LIDE-n) and prothrombin G20210A (Factor II mutation) produced similar results. The presence of blood group O was three-times more likely in the VITT-CVT *Definite* or *Probable* vaccinated group (OR = 3.45, 95% CI: 1.75–6.75, *p* < 0.001) compared with those with blood group A.

The demographic characteristics of vaccinated patients VITT-CVT defined as *Definite* or *Probable* and unvaccinated patients with CVT are presented in Table 3. No significant difference was observed in age and sex between them. More than 80% of the vaccinated VITT-CVT patients were classified as *Definite*.

**Table 3.** Characteristics of vaccinated VITT-CVT patients defined as *Definite* or *Probable* and unvaccinated patients with CVT.

	Vaccinated(*n* = 47)	Unvaccinated (*n* = 441)	*p*-Value
Age in years at CVT (SD)	46.2 (14.7)	47.4 (15.4)	0.60
Female (*n*/%)	37 (78.7)	305 (69.2)	0.23
COVID-19 vaccination (*n*/%)			
First (1st)			
ChAdOx1-S vaccine	47 (100.0)	0 (0)	–
Non-ChAdOx1-S vaccine^a^	0 (0)	0 (0)	–
Second (2nd)			
None	44 (93.6)	0 (0)	–
ChAdOx1-S vaccine	1 (2.1)	0 (0)	–
Non-ChAdOx1-S vaccine^b^	2 (4.3)	0 (0)	–
Third (3rd)			
None	47 (100.0)	0 (0)	–
ChAdOx1-S vaccine	0 (0)	0 (0)	–
Non-ChAdOx1-S vaccine^c^	0 (0)	0 (0)	–
VITT (*n*/%) for ChAdOx1-S vaccine			
Definite	38 (80.9)	0 (0)	–
Probable	9 (19.1)	0 (0)	–

*n*: sample size; SD: standard deviation; VITT: vaccine-induced immune thrombocytopenia and thrombosis.

^a^ Pfizer/BioNTech (0), Moderna (0), Johnson & Johnson (0), Sinovac (0), Other (0). ^b^ Pfizer/BioNTech (2), Moderna (0), Johnson & Johnson (0), Sinovac (0), Other (0). ^c^ Pfizer/BioNTech (0), Moderna (0), Johnson & Johnson (0), Sinovac (0), Other (0).

**Figure 2.**
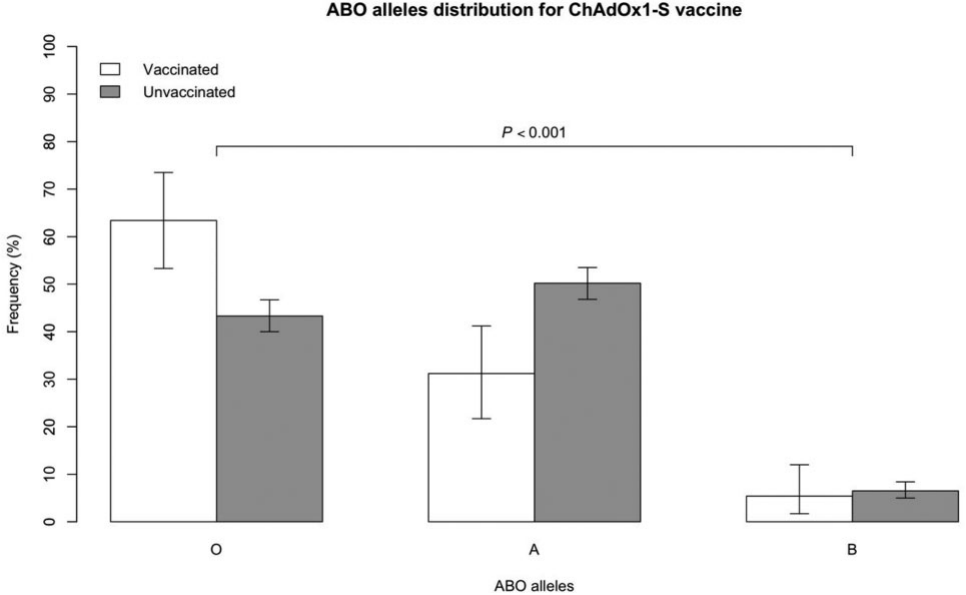
ABO allele distribution between ChAdOx1-S vaccinated and unvaccinated groups (analysis restricted to VITT-CVT defined as *Definite* or *Probable*^5^).

Further, to test whether ABO allele frequencies associated with ChAdOx1-S vaccination, the blood type allele distribution status among VITT-CVT patients defined as *Definite* or *Probable* is presented in Figure 2. ABO allele distribution can be more powerful and informative because it essentially doubles the sample size of the studied population.^16^ ChAdOx1-S vaccinated patients had 20.5% (95% CI: 43.3–63.8, *p* < 0.001) higher frequency of O allele compared with unvaccinated patients. Conversely, unvaccinated patients had higher frequency of the A allele 19.3% (95%CI: 8.8–29.8, *p* < 0.001) compared with vaccinated patients (Figure 2).

## Discussion

We show that blood group O is more prevalent among patients with VITT-CVT after ChAdOx1-S (Oxford-AstraZeneca) vaccination compared with unvaccinated cases in a well-characterised CVT population. The most common ABO allele among vaccinated patients was O, present in 63% (95% CI: 53–74) followed by allele-A present in 31% (95% CI: 22–41). Converse results were observed in the unvaccinated group with the most common ABO allele-A present in 50% (95% CI: 47–54) followed by the O-allele in 43% (95% CI: 40–47). Although our study is small, VITT-CVT post COVID-19 vaccination is rare and we present one of the largest cohorts recruited via two international ongoing studies.

There is increasing evidence for the involvement of blood group in CVT,^9^ either in terms of onset or outcome. We present data suggesting that blood group may also be involved in the development of CVT after vaccination. This finding not only may suggest an aetiological mechanism for CVT after a vector-based vaccine but may help to identify those more likely to be at risk. According to the European Medicines Agency,^17,18^ after the ChAdOx1-S vaccination, young people with wild type blood group O may experience immunosenescence, a negative immunological response that can result in blood abnormalities resembling disseminated intravascular coagulation. Blood group antigens play a direct role in infection through various mechanisms. Anti-A antibodies were protective against intracellular uptake of severe acute respiratory syndrome coronavirus 1.^19,20^ Blood group A with anti-A antibodies inhibited interaction between angiotensin converting enzyme-2-dependent cellular adhesion to angiotensin converting enzyme-2-expressing cells.^19^

Although we show a lower prevalence of blood group A for patients with VITT-CVT after ChAdOx1-S vaccination compared with unvaccinated cases and no significant differences observed for other known risk factors for CVT, our result must be interpreted with caution as the number of patients from the vaccinated group with ChAdOx1-S was limited. However, as CVT is a rare disorder, and vaccination-associated CVT is even rarer, our data present one of the largest such studies to-date. The population we recruited in both groups was well characterised using internationally agreed criteria for CVT and for vaccine-associated CVT. Other limitations include the possibility of the presence of specific confounders, namely, genetic or environmental factors that may have a role in modifying the vaccination effect. Additionally, side effects of ChAdOx1-S vaccination must be confronted knowing the vast number of doses that were administered over a short time.

## Conclusions

We document high prevalence of blood group O in patients with VITT-CVT after ChAdOx1-S Oxford-AstraZeneca vaccination. As ChAdOx1-S is the predominant vaccine of choice globally due to its ease of transportation and low cost, larger studies are urgently required to determine whether a complete ban on its use in young individuals with any blood group is too restrictive.

## Summary box

### What is known?

- COVID-19 patients with blood group A tend to have a higher risk of severe disease, while individuals with B or O blood groups are less represented among COVID-19 patients.
- CVT after administration of ChAdOx1-S (Oxford-AstraZeneca) has been associated within 28 days of vaccination.

### What is the question?

- This study aimed to determine whether blood group influences development of CVT after ChAdOx1-S vaccination.

### What was found?

- In this case–control study of 523 CVT patients from two international collaborative groups, we found that blood group O was more prevalent in the VITT-CVT group than in the unvaccinated pre-COVID-19 CVT group (43% vs. 17%, respectively, *p* < 0.001).
- Blood group A was less prevalent, though still high, in the vaccinated group compared with the unvaccinated group (47% vs. 71%, respectively, *p* < 0.001)
- Multivariate analysis with well-established CVT risk factors showed similar results.

### What is the implication for practice now?

- This study suggests that those with blood group O are more likely to be exposed to VITT-CVT after ChAdOx1-S vaccination regardless of well-established CVT risk factors such as gender.
- These results may lead governments to question whether ChAdOx1-S (Oxford-AstraZeneca) vaccine should be outright banned despite its high efficacy, cost-effectiveness and ease of handling.
- Those with non-O blood group may be suitable for the AstraZeneca COVID-19 vaccine, although larger studies are required to confirm our findings.

## Declarations

### Guarantor

PS.

### Provenance

Not commissioned; peer-reviewed by Julie Morris and Carmel Curtis.
